# Diagnostic accuracy of cerebrospinal fluid adenosine deaminase in detecting Tuberculous Meningitis

**DOI:** 10.12669/pjms.345.13585

**Published:** 2018

**Authors:** Aamir Habib, Zulfiqar Ali Amin, Syed Hassan Raza, Sobia Aamir

**Affiliations:** 1 Dr. Aamir Habib, MBBS. PNS Shifa Naval Hospital, Karachi, Pakistan; 2 Dr. Zulfiqar Ali Amin, FCPS(Medicine), FCPS(Oncology). PNS Shifa Naval Hospital, Karachi, Pakistan; 3 Dr. Syed Hassan Raza, MBBS. PNS Shifa Naval Hospital, Karachi, Pakistan; 4 Dr. Sobia Aamir, MBBS. PNS Shifa Naval Hospital, Karachi, Pakistan

**Keywords:** Meningitis, Tuberculous Meningitis, Adenosine Deaminase, Cerebrospinal Fluid

## Abstract

**Objective:**

To determine diagnostic accuracy of Cerebro Spinal Fluid (CSF) Adenosine De-Aminase (ADA) in detecting Tuberculous Meningitis (TBM) keeping CSF Polymerase Chain Reaction (PCR) for Mycobacterium Deoxy Ribonucleic Acid (DNA) as gold standard.

**Methods:**

This cross sectional validation study was conducted at Department of General Medicine of PNS Shifa Naval Hospital Karachi, Pakistan from Oct 2015 to Mar 2017 for a total duration of one and a half year. One hundred and thirty six patients were included. The diagnosis of TBM was based clinically on symptoms like fever, headache, altered mental state and signs of meningeal irritation with CSF findings of increased proteins, low glucose and lymphocytic pleocytosis. Lumbar puncture was done and approximately 4ml of CSF sample was withdrawn for analysis. Diagnosis of TBM was confirmed by doing CSF PCR test for mycobacterium tuberculosis DNA.

**Results:**

Total 136 patients were enrolled in this study. Mean age in our study was 47.09±12.80 years, whereas frequency and percentages of male and female patients was 102 (75%) and 34 (25%) respectively. The diagnostic accuracy, sensitivity, specificity, positive predictive value and negative predictive value of CSF ADA level in detecting TBM was 71.32%, 84.21%, 95.45%, 98.97% and 53.85% respectively.

**Conclusion:**

The study concludes that diagnostic accuracy of CSF ADA in detecting TBM is high which is proposed as an investigation to differentiate it from other causes of meningitis in places where PCR test is not available.

## INTRODUCTION

Meningitis is a medical, neurological and sometimes neurosurgical emergency that requires a multi-disciplinary approach. Most common types are bacterial, viral and Tuberculous Meningitis (TBM). TBM is more prevalent in the developing countries, especially in socioecomically low status countries.^1^ TBM accounts for about 10% of all cases of Tuberculosis (TB) in immune-competent individuals.^2^ According to World Health Organization (WHO) Global Health Observatory Database, TB accounts for 373 cases per 100,000 population in Pakistan.^3^

Jarvis et al in a study conducted in Cape Town, South Africa concluded that 28% cases of the total 4961 suspected cases were due to TBM.^4^ TBM can be fatal if not treated in time. If the diagnosis and treatment of TBM is delayed it can result in permanent neurological damage in 25% of patients.^5^

The diagnosis of TBM cannot be made on the basis of clinical findings alone because almost all types of meningitis present with more or less the same findings. Diagnosis requires observation of tubercle bacilli in the Ziehl-Neelsen staining of the Cerebro Spinal Fluid (CSF) but it lacks sensitivity. Similarly CSF culture is also diagnostic but the results are often too late, takes 4-6weeks.^6^ Computerized Tomography (CT) Scan and Magnetic Resonance Imaging (MRI) brain lacks specificity as far as TBM is considered. Newer techniques that involve amplification of bacterial Deoxy Ribonucleic Acid (DNA) by Polymerase Chain Reaction (PCR) can provide a reliable diagnosis of TBM but in developing countries with limited resources it cannot be easily done. Similarly diagnosis can be confirmed by CSF Gene expert which is PCR based test which gives result in two days and is the standard of care in the diagnosis of tuberculosis and it gives information about rifampicin resistance as well. CSF analysis in TBM shows elevated proteins and low glucose with lymphocytic pleocytosis but patients with Herpes encephalitis, cryptococcal meningitis, brucellosis and neurosyphilis also exhibit more or less same picture so there arise difficulty in differentiating TBM from these causes of meningitis. Therefore an inexpensive diagnostic tool for TBM is required which can easily differentiate TBM from other causes.

Adenosine deaminase (ADA) is an enzyme which is involved in purine metabolism in which it is responsible for conversation of adenosine and deoxyadenosine to inosine and deoxyinosine respectively resulting in release of ammonia. In TBM tubercle bacilli results in cell mediated immune response which causes release of ADA by T cells but it is also released in other conditions which involve activation of cell mediated immunity.

International studies have been performed to evaluate the diagnostic and predictive value of CSF ADA in TBM patients. Agarwal et al in a hospital based study showed that ADA estimation in CSF with a cut-off value of 10U/L is simple, cost effective and less time consuming method for making a diagnosis of TBM, especially when there is a difficulty in differentiating whether it is due to tuberculous etiology or non tuberculous etiology.^5^ Nepal et al recommended that CSF ADA activity in TBM (11.1±2.03 IU/L) is simple, inexpensive, sensitive and specific test for tubercular disease and it need to be performed before sending the cumbersome and expensive procedures like culture and PCR for TB diagnosis.^7^ Similarly Rana et al in a study showed that using a cut-off value of >10U/L, CSF ADA had a sensitivity of 92.5% and specificity of 97% for diagnosis of TBM and concluded that CSF ADA is a more sensitive indicator than PCR for TBM diagnosis.^8^ Moghtaderi et al however showed that a positive value of ADA activity cannot confirm TBM but it may lead physicians to treat patients earlier before the confirmatory diagnostic reports will be received. A cut-off value of 10.5U/L was considered highly sensitive and specific.^9^ Local studies have not been done to show the diagnostic value of CSF ADA in TBM patients. The purpose of this study was to determine diagnostic accuracy of CSF ADA in detecting TBM and initiating therapy on its basis.

## METHODS

This cross-sectional study was conducted at PNS Shifa Naval Hospital, Karachi from October 2015 to March 2017 over a period of one and a half year. After approval from the hospital’s ethical and research committee, informed written consent was obtained from the patient or his next of kin in case patient lacked decision making capacity, after explaining the purpose and benefits of study. All indoor patients meeting the inclusion criteria were included in the study aged between 12 to 85 years. The diagnosis of TBM was based on symptoms like fever, headache and altered mental state and signs like neck stiffness and positive kerning’s sign with CSF routine examination (R/E) findings of increased proteins, low glucose and lymphocytic pleocytosis. Diagnosis of TBM was confirmed by doing CSF PCR test for Mycobacterium TB DNA. Patients who were not willing for initial diagnostic tests were excluded.

All patients were subjected to detailed history and examination. Infarct, hemorrhage and Space occupying lesion (SOL) brain was ruled out on CT scan brain. The fundus was examined to rule out papilloedema. Within one hour of admission, Lumber puncture was done and approximately 4ml of CSF sample was withdrawn under strict aseptic technique and was sent to specified laboratory of the hospital. CSF sample about 2ml was used for cell count, biochemistry and smears for gram staining and staining for AFB and 1ml for ADA levels estimation under supervision of pathologist. ADA activity in CSF was estimated according to the method of Galanti and Giusti colorimetric method and was expressed as U/L. Remaining sample of CSF was used for PCR for MTB DNA for confirmation. PCR machine used was smart cycler which determines real time pcr which involves DNA extraction and amplification. PCR was used as gold standard because culture results takes 2-3 weeks to come and determination of AFB in CSF lacks sensitivity.

Frequencies and percentages were calculated for qualitative variables like gender, true positive and true negative. For quantitative variables like age, mean and standard deviation was used. Effect modifiers like age and gender were controlled by stratification. All results were presented as tables and graphs. Post stratification chi-square test was applied. p value ≤ 0.05 was considered statistically significant.

## RESULTS

A total 136 patients were enrolled in the study. Mean age of study population was 47.09+12.80 years with ranges from 20-85 years. Out of 136, 102 (75%) were males, and 34 (25%) were females with a M:F ratio of 3:1 but patients were randomly selected who visited hospital during the study time frame. A 2x2 table was constructed for comparison, given in Table-I. Diagnostic accuracy, sensitivity, specificity, positive predictive value and negative predictive value of CSF ADA level in detecting TBM is given in Table-II.

**Table-I:** Comparison of CSF ADA level (u/l) with CSF PCR as Gold Standard.

CSF ADA level (u/l)	CSF PCR		Total

	Positive	Negative	
Positive (>10 u/l)	96	1	97
Negative (<10 u/l)	18	21	39

Total	114	22	136

**Table-II:** Clinical Efficacy of CSF ADA level (u/l).

Sensitivity (%)	84.21
Specificity (%)	95.45
Positive Predictive Value (%)	98.97
Negative Predictive Value (%)	53.85
Diagnostic Accuracy (%)	71.32

## DISCUSSION

TBM is a common disease in socioeconomically low status countries. In India every year about 0.5 million patients die because of tuberculosis, 8.3% of which is occurring in children only. Emergence of resistance to multiple drugs in tuberculosis and occurrence of Acquired Immuno-Deficiency Syndrome (AIDS) along with TB has further worsen the disease outcome. Among extrapulmonary TB, TBM is the most serious form with incidence of 7 to 12% of all cases of TB in developing countries. If there is any delay in diagnosing of the disease or starting of effective treatment than it can result in permanent neurological damage in up to 25% of cases resulting in poor prognosis. The methods available for the diagnosis of TBM were checked but all were found to be of low sensitivity and specificity.^1^

Many methods are available for the diagnosis of TB. Light microscopy is used for the detection of Acid Fast Bacilli (AFB) in a smear throughout the world and the rate of detection is 30-40%.^10^ Culturing on Lowenstein-Jensen (LJ) medium has a higher sensitivity than microscopy but it requires many weeks for incubation. A number of nucleic acid amplification tests are also available which includes GenProbe amplified Mycobacterium tuberculosis direct test, CobasAmplicor test, Roche Amplicor MTB test, the BD-Probe Tec test and Abbott LCx test. They are effective for detecting TB but their cost is high that’s why they are not routinely used especially in developing countries. Culture and PCR both can be used as gold standard but culture is time consuming and PCR test is costly and not readily available. CSF ADA test is readily available, cost effective and less time consuming.

ADA is basically an enzyme with very high levels in T-lymphocytes where its role is in the differentiation of lymphoid cells. During lymphocytes antigenic responses ADA levels increases. Defects in cell mediated and humoral immunity is associated with low levels of ADA making the patient prone to many opportunistic infections.^1^ In TBM tubercle bacilli results in Cell Mediated Immune Response (CMI) which causes T cells to release ADA. Many studies have also shown that ADA levels can be used to differentiate TBM from non tuberculous cause.^11^

Rana et al^8^ in their study observed that the frequency and percentage of male and female patients were 22(40.7%) and 32(59.3%) respectively. While in our study, frequency and percentages of male and female patients was 102 (75%) and 34 (25%). This difference in demography could be due to government hospital and secondly most of our dependent population lives in official accommodation without families. In our study, the diagnostic accuracy of cerebrospinal fluid adenosine deaminase (ADA) level u/l in detecting TBM with CSF PCR was having the sensitivity and specificity of 84.21% and 95.45% with a CSF ADA cutoff level of >10. Similarly Rana et al.^8^ in their study showed that using a cut-off value of >10U/L,CSF ADA had a sensitivity of 92.5% and specificity of 97% for diagnosis of TBM and is more sensitive indicator than PCR which he used as gold standard.

Saini et al.^12^ in a study conducted in India showed that out of 143 cases, 40 were TBM and 103 were non-TBM. The mean ADA levels in TBM was 17.18 and in non-TBM was 6.33. Using a cutoff level of >10 CSF ADA has a sensitivity of 92.5% and specificity 89.32%. He used culture and PCR test as gold standard. Li et al.^13^ in a Chinese study concluded that CSF ADA in TBM patients was significantly higher than in non-TBM patients using CSF culture as gold standard. CSF ADA was reduced with anti-TB treatment also. So CSF ADA cannot only be used for diagnosis but also for treatment response. Chacko et al.^14^ in his study included fifty four adult patients with suspected TBM and 37 controls. Using a cutoff level of >10 CSF ADA has a sensitivity of 92.5% and specificity of 97% and concluded that CSF ADA is a rapid and inexpensive test in diagnosis of TBM even more sensitive than AFB smear and culture.

CSF ADA average cost is Rs. One thousand and CSF PCR cost is around Rs. Five thousand. CSF ADA test is readily available while PCR test is not available everywhere.

## CONCLUSION

The study concludes that diagnostic accuracy of CSF ADA in detecting TBM is high (71.32%) which proposed it as an investigation to differentiate it from other causes of meningitis in places where PCR test is not available. As in Pakistan, PCR for Mycobacterium Tuberculosis DNA in CSF is not available everywhere and therefore CSF ADA can be readily available parameter in this regard. This test is cheap and results can be available early as compared to CSF PCR test so it can be used for detecting TBM.

### Authors’ Contribution

**AH** conceived, designed the study, did data collection.

**ZAA** did editing and finally approved manuscript.

**SHR** did statistical analysis, manuscript writing.

**SA** did editing, manuscript writing.
